# Endothelial cell-surface tissue transglutaminase inhibits neutrophil adhesion by binding and releasing nitric oxide

**DOI:** 10.1038/s41598-017-16342-0

**Published:** 2017-11-23

**Authors:** Thung-S. Lai, Robert A. Lindberg, Hua-Lin Zhou, Zishan A. Haroon, Mark W. Dewhirst, Alfred Hausladen, Y.-L. Juang, Jonathan S. Stamler, Charles S. Greenberg

**Affiliations:** 1 Institute of Biomedical Science, Mackay Medical College, New Taipei City, Taiwan, ROC; 2 North Carolina Biotechnology Center, Research Triangle Park, Triangle Park, NC United States; 3 Carolina Institute for Nanomedicine, University of North Carolina, 1079 GMB, CB7295 Chapel Hill, NC United States; 4 Department of Radiation Oncology, Duke University Medical Center, Durham, NC United States; 5 Institute for Transformative Molecular Medicine, Case Western Reserve University and University Hospitals Cleveland Medical Center, Cleveland, OH USA; 6 Harrington Discovery Institute, University Hospitals Cleveland Medical Center, Cleveland, OH USA; 7 Hematology/Oncology, Medical University of South Carolina, Charleston, SC United States

## Abstract

Nitric oxide (NO) produced by endothelial cells in response to cytokines displays anti-inflammatory activity by preventing the adherence, migration and activation of neutrophils. The molecular mechanism by which NO operates at the blood-endothelium interface to exert anti-inflammatory properties is largely unknown. Here we show that on endothelial surfaces, NO is associated with the sulfhydryl-rich protein tissue transglutaminase (TG2), thereby endowing the membrane surfaces with anti-inflammatory properties. We find that tumor necrosis factor-α-stimulated neutrophil adherence is opposed by TG2 molecules that are bound to the endothelial surface. Alkylation of cysteine residues in TG2 or inhibition of endothelial NO synthesis renders the surface-bound TG2 inactive, whereas specific, high affinity binding of S-nitrosylated TG2 (SNO-TG2) to endothelial surfaces restores the anti-inflammatory properties of the endothelium, and reconstitutes the activity of endothelial-derived NO. We also show that SNO-TG2 is present in healthy tissues and that it forms on the membranes of shear-activated endothelial cells. Thus, the anti-inflammatory mechanism that prevents neutrophils from adhering to endothelial cells is identified with TG2 S-nitrosylation at the endothelial cell-blood interface.

## Introduction

The physical interaction between neutrophils and activated endothelium is among the earliest events in inflammation^1^. Initial capture and rolling of neutrophils are primarily mediated by glycoproteins called selectins^2^. E-selectin and P-selectin on the luminal surface of endothelial cells bind to L-selectin on the neutrophil surface^2^. The firm adhesion of neutrophils to endothelium is then carried out by the leukocyte β_2_ integrins LFA-1 and Mac-1 (heterodimeric surface proteins) and their endothelial counter-receptor molecule, intercellular adhesion molecule-1 (ICAM-1)^3,4^. The expression of both neutrophil and endothelial cell adhesion molecules is up-regulated by tumor necrosis factor-α (TNFα)^5,6^.

Nitric oxide (NO) bioactivity can influence both the release of inflammatory cytokines and the expression of cell adhesion molecules, and it is recognized that production of NO by endothelial cells regulates neutrophil adhesion *in vivo* ^6–11^. Endothelium-derived NO that diffuses luminally must avoid inactivation by hemoglobin in the blood stream—a problem solved by preserving NO bioactivity in the form of S-nitrosothiols (SNOs), which are impervious to heme scavenging^12^. Under this model, NO bioactivity in endothelium and RBCs may be exchanged through sequential transnitrosylation reactions^13^ involving SNO-proteins spanning the RBC-endothelial cell unit^12,14^. This model is supported strongly by the observation that genetically-induced depletion of RBC SNO impairs endothelial relaxations^15^. In addition, transgenic animals with increased levels of SNOs in red blood cells are hypotensive, reflecting connectivity between RBC and vessel wall^14,16^.

Tissue transglutaminase (TG2) is ubiquitously distributed^17^, with the highest expression found in endothelial cells (ECs)^17,18^. TG2 is localized to cell-surfaces (where it is membrane-associated), cytoplasm and nucleus of cells^17^. Through an unconventional mechanism, a considerable amount of TG2 is secreted into the extracellular matrix (ECM) and involved in ECM stabilization and cell adhesion^17,19,20^. TG2 possesses classical transamidation activity as well as non-enzymatic activities^17,21^. The transamidation activity of TG2 is regulated by calcium ions, GTP, NO and redox state^22,23^. TG2 is enzymatically inactive in in the extracellular milleu^23^, which may be attributed to its oxidation^24^. We have previously demonstrated that S-nitrosylation may also inhibit TG2’s activity^22^. S-nitrosylation also regulates TG2 externalization^19,20^, potentially implicating NO in the mechanism of oxidative inactivation. TG2 is S-nitrosylated in young aorta but not in aged aorta^25^ and deficient S-nitrosylation has been linked to vascular stiffness^26,27^. Extracellular TG2 has also been implicated in Celiac disease, cancer, and fibrotic disorders^28,29^. These *in vitro* and *in vivo* data argue for potential roles of non-canonical TG2 activity in human health and disease that may be regulated by NO/redox.

TG2 has 18 free sulfhydryl (−SH) groups, several of which are contained within S-nitrosylation motifs^22,30^, consistent with our report that it undergoes poly-S-nitrosylation^22^. We therefore considered the possibility that TG2 may provide a reservoir of NO bioactivity at the endothelial cell surface. Here we show that anti-inflammatory activity of endothelium-derived NO at the endothelial-blood cell interface is conveyed by SNO-TG2.

## Results

TG2 is bound constitutively to endothelial surfaces at the blood interface and transcriptionally up-regulated by TNFα^17,31^. We sought a model system to examine the effect of surface-bound TG2 on polymorphic neutrophil (PMN) adherence and to test the influence of NO under inflammatory condition. Confluent HUVEC cells were treated with increasing concentrations of TNFα and and the expression of various cell adhesion molecules were investigated. After 5 hours of TNFα treatment, cells remains viable as examined by morphology and cell viability assays (Supplemental Fig. 1). The expression of cell adhesion molecules including E-selectin, ICAM-1, and P-selectin were found to be upregulated by TNFα treatment (Supplemental Fig. 2). By contrast, TNFα did not alter the surface expression of TG2, which was found to be constitutively expressed on the luminal surface of HUVECs monolayers (Supplemental Table 1).

To directly visualize surface-bound TG2, we added glutathione S-transferase (GST)-TG2 (or active site mutant GST-TG2/C277A) to confluent HUVEC monolayers. Isolation of total cytosolic and membrane fractions confirmed that both GST-TG2 and GST-C277A/TG2 were bound to the surface of endothelial cells (Supplemental Figs 3 and 4). GST-TG2/C277A binding increased following TNFα treatment of HUVECs. GST-TG2-treated samples showed multiple high molecular weight bands, likely reflecting crosslinking by canonical TG2 activity (Supplemental Fig. 3). Endogenous TG2 (80 kDa) is predictably distributed between cytosol and membrane (Supplemental Figs 3 and 4).

**Figure 1:**
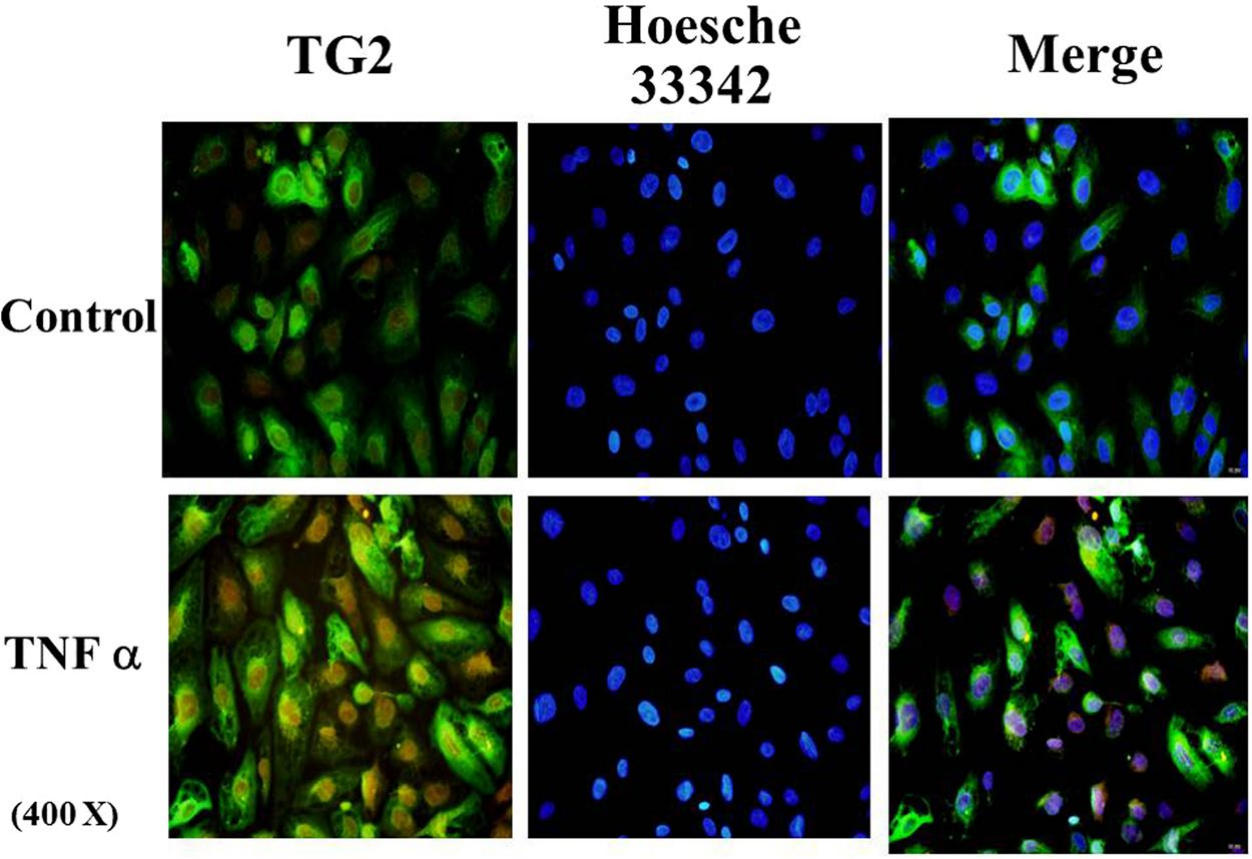
Immunofluorescent staining of TG2 in TNFα-treated HUVEC cells. HUVEC cells were grown and treated with or without TNFα (10 ng/ml) for 5 hours. The cells were non-permeabilized, fixed with 4% paraformaldehyde and stained with mouse monoclonal anti-TG2 (TG100) followed by donkey anti-mouse IgG (Fitc conjugated, shown in Green). The fluorescent signal was visualized using fluorescent microscopy. The nuclei were stained with Hoesche 33342 (shown in blue).

**Figure 2:**
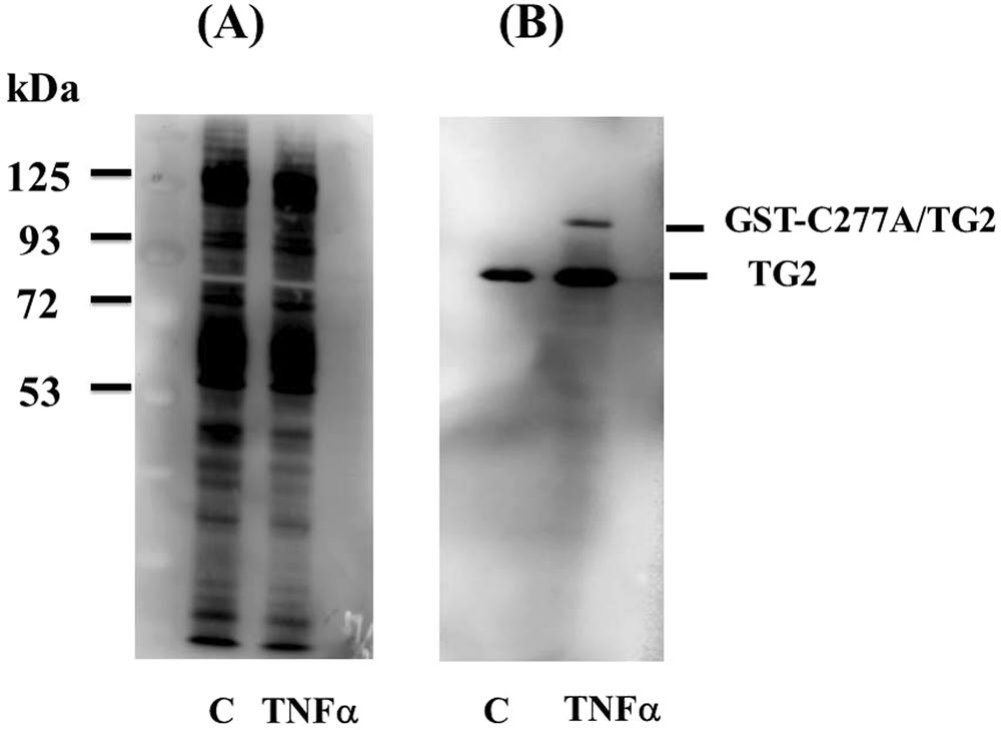
Cell surface biotinylation of cell surface proteins identifies endogenous and exogenous TG2. HUVEC confluent monolayers were incubated with GST-TG2/C277A (50 nM) during the last hour of a 5 hr TNFα (10 ng/ml) treatment. Total cell lysates including membrane fractions were isolated after cell surface proteins biotinylation as described under Materials and Methods. **(A)** Total cell lysates (15 μg) were separated by SDS-PAGE and transferred to PVDF membrane. Blots were developed using Streptavidin-HRP and bands were visualized using ECL chemiluminescence as described under Materials and Methods. (**B)** Streptavidin bead pull-down of biotinylated proteins from total cell lysates derived from (**A**) were performed as described under *Materials and Methods.* The bound biotinylated proteins were eluted from streptavidin-beads by incubating with SDS-PAGE loading buffer with heating at 95 °C for 4-min. Half of the eluted samples were loaded on a SDS-PAGE and transferred to PVDF membrane. Blots were developed using TG2 mouse monoclonal antibody (cub7402) and goat vs mouse IgG conjugated with HRP and bands were visualized using ECL chemiluminescence.

Additional experiments were carried out to validate the surface localization of TG2. TG2 was found to express on HUVEC surfaces using non-permeabilized cell staining and fluorescent visualization (Fig. 1). Cell surface biotinylation (labeled with a cell-impermeant Biotin-XX sulfosuccinimidyl ester) followed by streptavidin pull-down also demonstrated labeling of endogenous and exogenous TG2 (Fig. 2).

We determined that specific TG2 binding sites could be saturated by the addition of TG2. Using an ELISA assay to measure surface-bound TG2, the addition of 50 nM TG2/C277A resulted in a two-fold increase in surface binding (mean relative OD_405 nm_ = 0.053 ± 0.028 *vs*. 0.105 ± 0.017; n = 4, p < 0.05) (Supplemental Table 1). The binding of TG2/C277A did not alter either the constitutive or TNFα-induced expression of ICAM-1 as measured by flow cytometry (Supplemental Fig. 5).

**Figure 3:**
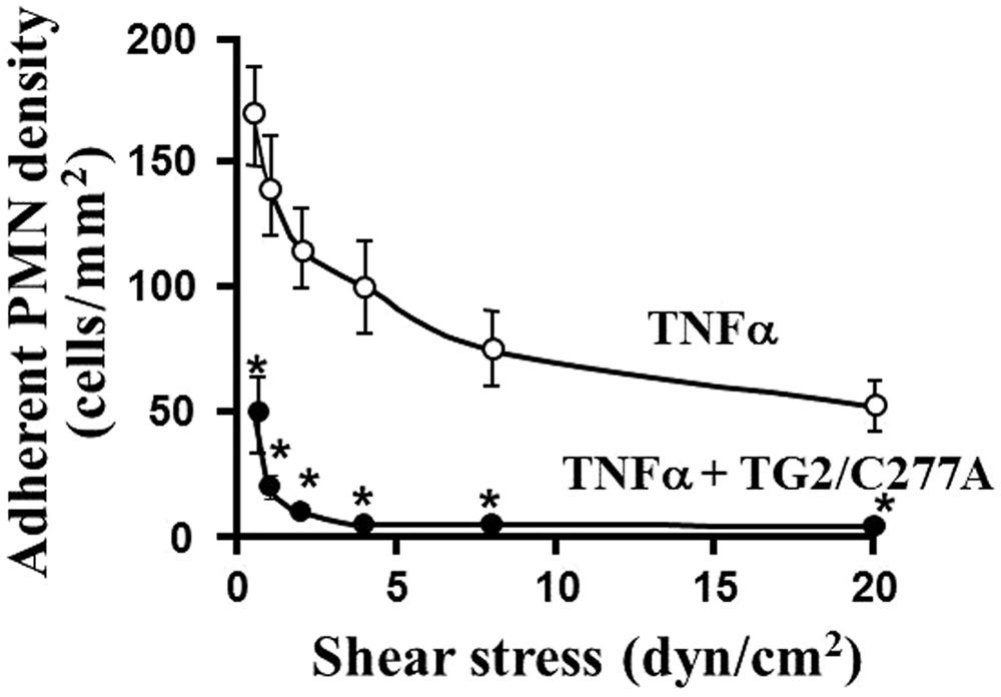
TG2/C277A inhibits neutrophil adhesion to TNFα-activated endothelium. HUVEC monolayers were treated with TG2/C277A (10 nM) during the last hour of a 5 hr TNFα (10 ng/ml) treatment. After infusion of neutrophils (PMN), cultures were exposed to shear stress at the values indicated, and adherent PMNs were then counted. Data are presented as means ± SEM. TNFα, n = 5; TNFα/TG2/C277A, n = 4. *p* < 0.05, TNFα vs. TNFα/TG2/C277A at all shear stress values. The statistical significance at each shear stress was analyzed using student t-tests. Asterisks denote significant differences. The p-values at each shear stress are as followings: 0.5 dyn/cm^2^, p = 0.023902; 1 dyn/cm^2^, p = 0.010764; 2.0 dyn/cm^2^, p = 0.007297; 4.0 dyn/cm^2^, p = 0.008728; 8.0 dyn/cm^2^, p = 0.015503; 20 dyn/cm^2^, p = 0.010764.

As extracellular TG2 is enzymatically inactive^23^, we employed the enzymatically- inactive mutant, TG2/C277A, to examine the role of TG2 on PMN binding to endothelium. Recombinant TG2/C277A was added to HUVEC monolayers after the activation with TNFα, and PMN adherence was then monitored under physiological shear (to simulating blood flow). TG2/C277A inhibited both the initial binding and the attachment (>30 sec) of PMNs (Fig. 3) with complete inhibition of binding seen at 100 nM TG2/C277A (Supplemental Fig. 6), and inhibition was maintained across the physiological range of shear rates (τ_ω_ = 0.4–20 dyn/cm^2^; p < 0.05) (Fig. 3). There was no difference in neutrophil rolling in the presence or absence of TG2/C277A (PMN roller flux = 16 ± 8.5 vs. 19 ± 7.8 at τ_ω_ = 1 dyn/cm^2^, respectively. n = 3; p < 0.05), indicating that the inhibition of neutrophil adhesion was due to an effect on PMN attachment (data not shown). Interestingly, active TG2 was found to have less anti-adhesive effects than the mutant (Supplemental Fig. 7). Thus, the activity of extracellular TG2 reduces its anti-PMN binding ability, and TG2/C277A binding to TNFα activated endothelial cells inhibits PMN binding, independent of TG2’s transamidation activity.

**Figure 4:**
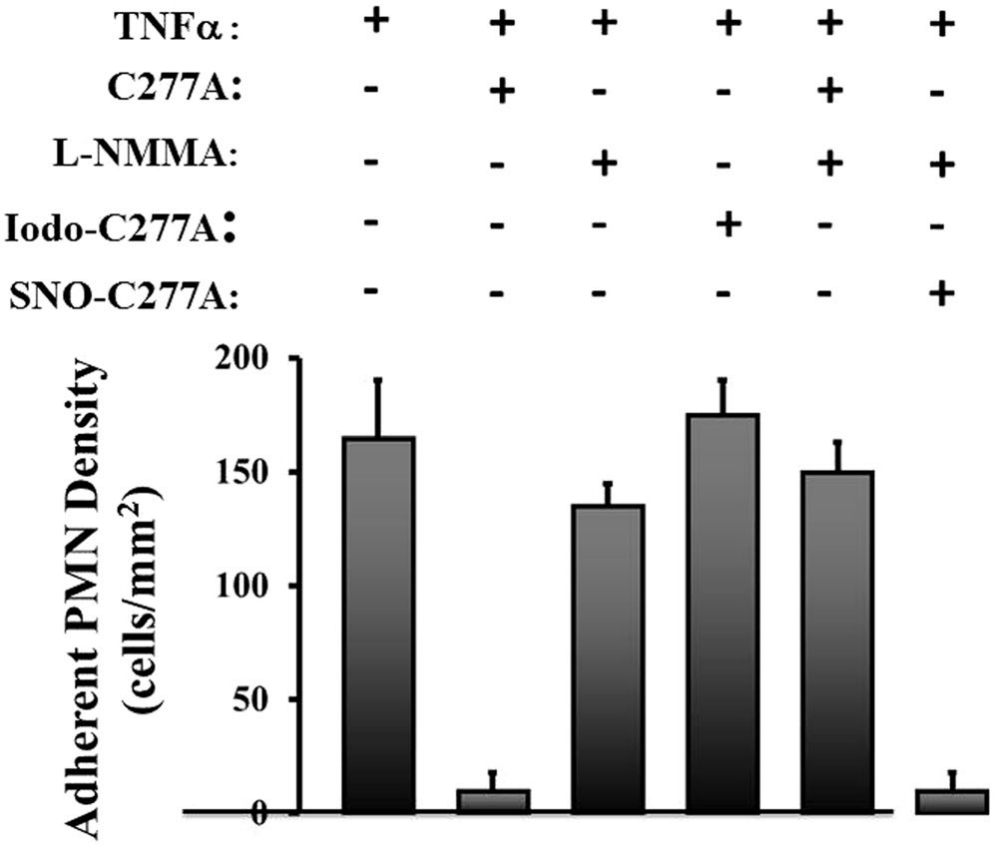
Inhibition of PMN adhesion to endothelial cells is NO dependent. Inhibition of NO synthase (NOS) with L-NMMA prevents inhibition by TG2/C277A of PMN adhesion to endothelial cells pretreated with TNFα, and exogenously S-nitrosylated TG2/C277A inhibits PMN adhesion to TNFα-activated PMNs in the presence of L-NMMA (150 μM). Alkylated TG2/C277A (iodo-TG2/C277A) also prevents inhibition of PMN adhesion. TNF-activated endothelial cells were treated with TG2/C277A, iodo-TG2/C277A or S-nitrosylated TG2/C277A (50 nM, 1 hr) and exposed to flow for 20 min prior to infusion of PMNs. Wall shear stress (τ_ω_) = 1 dyn/cm^2^. Data are presented as means ± SEM, n ≥ 3. *p < 0.05 vs. TNFα. Statistical analysis was performed with a single-factor ANOVA using shear stress = 1.0 dyn/cm^2^ in all groups: TNFα vs. TNFα/C277A/TG2 (p = 0.002); TNFα/TG277A vs. TNFα/L-NMMA/C277A/TG2 (not nitrosylated) (p = 0.003); TNFα/L-NMMA/SNO-C277A/TG2 vs. TNFα/L-NMMA/C277A/TG2 (not nitrosylated) (p = 0.00044).

We have shown previously that stimulation of endothelial nitric oxide synthase (eNOS) within aortic endothelial cells (by treatment with Ca^2+^ ionophore) results in the S-nitrosylation of exogenous TG2^22^. Similarly, following addition of TG2/C277A to HUVECs, activation of eNOS by shear stress resulted in the S-nitrosylation of about 14% of exogenous TG2/C277A thiols (comparable to results with aortic endothelial cells^18^). In our current work, shear stress activation of eNOS led to a large decrease in the adherence of PMNs, and this effect was abrogated by treatment with the NOS inhibitor L-NMMA, whereas addition of S-nitrosylated TG2/C277A inhibited PMN adherence in the presence of L-NMMA (Fig. 4). Further, pre-treatment of TG2/C277A with the thiol alkylating agent iodoacetamide, which would prevent subsequent S-nitrosylation, abrogated the decrease in adherence consequent upon activation of eNOS (Fig. 4). Thus, the ability of TG2 to inhibit PMN adherence to endothelial cells may be dependent upon S-nitrosylation of TG2, and the active site (C277) of TG2 is not involved.

**Figure 5:**
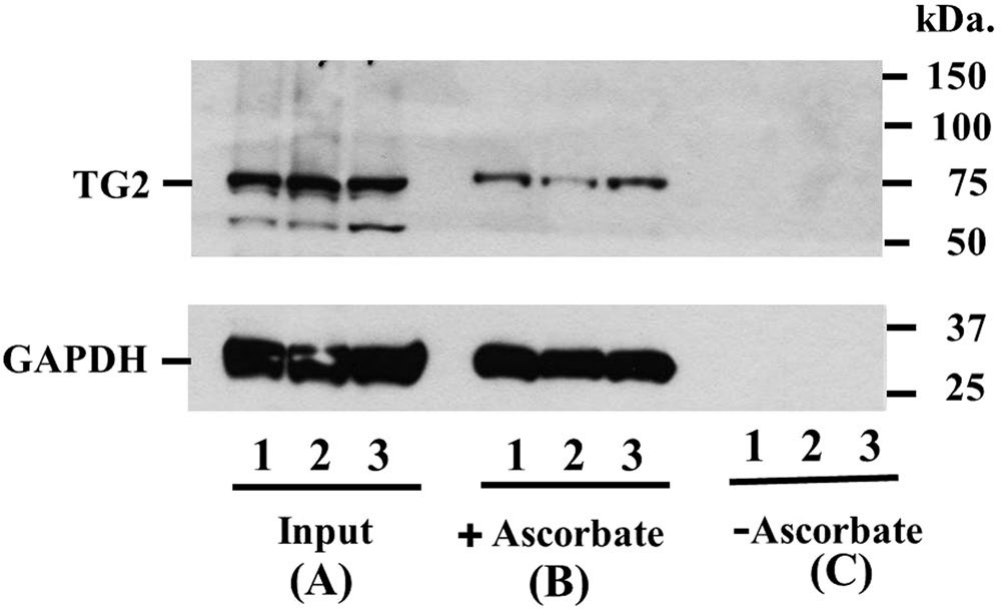
Endogenous TG2 is robustly S-nitrosylated in Mouse Tissues. SNO-TG2 isolated from three separate mouse kidneys (designated as 1, 2 and 3). 10 μg samples were loaded on to each lane for immunoblot analysis. (**A**) 0.5% of total input (**B**). Proteins eluted from ascorbate treated samples (+ascorbate) = SNO-proteins. (**C**). Proteins eluted from untreated samples (−ascorbate) = controls. SNO-modified TG2 was eluted from thiopropyl-Sepharose beads and analyzed by immunoblots using mouse monoclonal antibody against TG2 (cub7402, ThermoFisher Scientific) as described under *Materials and Methods*. GAPDH, a prototypic SNO-modified protein (detected using rabbit monoclonal antibody Abcam, Ab181602) is included as a control.

Finally, we determined that SNO-TG2 is present *in vivo*, as demonstrated following resin-assisted capture (SNO-RAC) of SNO-TG2 from three different mouse kidney extracts (Fig. 5). Scanning densitometry of SNO-TG2 demonstrates that almost 50% (44 ± 15%) of TG2 is S-nitrosylated *in situ* (Fig. 5). As a further control, we show that GAPDH, a prototypic SNO-protein^32^, is readily detected under these conditions.

## Discussion

Endothelial NO synthase is localized to the plasma membrane to enable NO production at cell-cell interfaces. In conventional models, NO diffuses luminally to exert anti-inflammatory activity by preventing neutrophil adhesion. Accumulating evidence indicates, however, that NO bioactivity *in vivo*, including anti-inflammatory activity, is conveyed mainly through SNOs, which entails S-nitrosylation of key target proteins^33–35^. Under this model, NO bioactivity in the endothelium would transfer to blood cells through coupled equilibria involving SNOs^13^. Endothelial cell surfaces (and adjacent ECM) are in fact rich in protein thiols, raising the idea that SNOs at the cell surface may provide a source of NO bioactivity. Here we show that SNO-TG2 is readily detectable in highly vascularized tissues (Fig. 5). We also show that TG2 serves as a co-factor for endothelium relaxing factor (EDRF)/NO bioactivity, specifically, in maintenance of a non-adherent endothelial surface.

Endothelial cells are among richest cellular sources of TG2^18^. TG2 is concentrated in or at the cytoplasm, nucleus and plasma membrane where it contributes reducing equivalents (18 SH groups per protein)^17^. TG2 is also secreted on to endothelial surfaces, where it functions as a co-receptor for fibronectin^36^, and into the ECM (where it also binds fibronectin). TG2 is therefore a potential candidate for modulation of a variety of endothelial cell functions. Here we describe a novel and unanticipated role for TG2 in transducing the anti-adhesive properties of cytokine-activated endothelial cells.

Extracellular TG2 is oxidatively inactivated *in vivo* and does not mediate transamidation reactions^23^. Therefore to mimic physiological conditions, we used an active site mutant, TG2/277A^23^. Because TG2/C277A is devoid of enzymatic activity, it cannot alter cell adhesion forces directly. Using non-permeabilization fixation staining and cell surface biotinylation techniques, we confirmed that TG2 is expressed on the surface of control and TNFα–stimulated cells (Figs 1 and 2), and that surface-bound C277A/TG2 (Supplemental Figs 3 and 4) retains ability to inhibit neutrophil adhesion. We further showed that the anti-adhesive properties of TG2 are dependent on both cysteine thiols and NO. In particular, inhibition of eNOS prevented the inhibition by TG2 of neutrophil adhesion to TNFα-activated endothelial cells. We also showed that S-nitrosylated TG2/277A recapitulated the anti-inflammatory property of endothelial surfaces. It has been previously reported that TG2 S-nitrosylation, protects against vascular stiffness^25^. However, this function is critically dependent on the enzymatic activity of intracellular TG2. Thus, S-nitrosylated TG2 is formed both intracellularly and extracellularly to regulate vascular stiffness and inflammation, respectively.

The regulation of extracellular TG2’s enzymatic activity has been the subject of intense interest since TG2 is involved in the pathogenesis of a number of inflammatory disorders, including arteriosclerosis^25^, fibrosis and Celiac disease^21,29^. In Celiac disease, TG2’s enzymatic activity is responsible for the deamidation of gluten peptides, which trigger an inflammatory T cell response, ultimately resulting in the destruction of the small-bowel mucosal architecture^37^. Circulating IgA in the blood of Celiac patients collaborates with thioredoxin to activate extracellular TG2^24,38^. By contrast, in our model of vascular inflammation, the activity of extracellular TG2 was dependent on its S-nitrosylation state and inactivation by NO was associated with enhanced anti-inflammatory properties.

The current paradigm regarding leukocyte-endothelium interactions indicates that initial capture and rolling of leukocytes is mostly mediated by low affinity selectins^39^, whereas firm adhesions are mediated by ICAM-1 and integrins. Initial low affinity interactions are subsequently strengthened as a result of leukocyte activation. In our leukocyte adhesion model, E-selectin, P-selectin and ICAM-1 are all induced after TNFα treatment (see Supplemental Fig. 2). However, cell surface C277A/TG2 did not affect leukocyte rolling. Inhibition by SNO-TG2 of firm adhesion would be consistent with the known effect of endothelial NO to inhibit ICAM-1 expression^40^. Further studies are needed to elucidate the exact mechanism by which SNO-TG2 inhibits leukocyte adhesion.

Our findings demonstrate a role for TG2 in transducing the anti-inflammatory properties of NO produced by shear- and cytokine-activated endothelial cells and show that NO bioactivity identified with eNOS is, at least partly, juxta-membrane SNO-TG2. Thus, it would appear that endothelial NO does not diffuse into the lumen to inhibit neutrophils, but rather concentrates on the luminal surface to prevent neutrophil attachment. These data expand the perspective on the co-localization of NO sources and targets to include endothelial NOS and extracellular surface ligands^41^, and emphasize that NO bioactivity may derive from sources other than NOS. Inasmuch as the anti-adhesive properties of NO are mediated by S-nitrosylation of neutrophil proteins (e.g. NFκB), delivery of NO bioactivity by SNO-TG2 may occur via sequential transnitrosylation reactions, by analogy to the coupled equilibria between SNOs in RBCs and the vascular wall that dispense NO bioactivity^12,13^.

We showed previously that activating eNOS within endothelial cells by increasing intracellular Ca^2+^ levels with Ca^2+^ ionophore leads to S-nitrosylation of TG2 and here extend our finding to shear stress, which similarly activates eNOS by increasing intracellular Ca^2+^ levels. We have also shown that Ca^2+^ can effect NO group release from SNO-TG2 by an allosteric mechanism^22^, and it has also been reported that S-nitrosylation of TG2 may regulate its distribution between intracellular and extracellular loci^20^. Thus, our data raise the intriguing possibility that regulation of NO bioactivity at the endothelial-blood interface may depend on whether Ca^2+^ originates from extracellular or intracellular pools: whereas intracellular Ca^2+^ will activate eNOS to S-nitrosylate TG2^42^, Ca^2+^ released extracellularly (e.g. by adhering neutrophils) might release NO groups from surface TG2. SNO-TG2 may also serve to prevent adherence of platelets and RBCs, emphasizing that SNOs within membranes have additional signaling functions^43^.

Externalization of TG2 by aortic endothelial cells (and by inference amounts of surface bound SNO-TG2) was decreased by high levels of sustained laminar shear stress (18 dynes/cm^2^ for 16 hr)^19^. By contrast, brief periods of laminar shear stress (<1 min under each shear stress range from 0.4 to 20 dynes/cm^2^) lead to robust S-nitrosylation of externalized TG2. TG2 localization, activity and function are thus predicted to vary in vascular health and disease and to reflect the S-nitrosylation state at the cell-cell interface.

## Experimental procedures

### Reagents

Reagents and materials were obtained from the following suppliers: Medium 199 (M-199, Earle’s salts or Hank’s salts), antibiotic/antimycotic (pen-strep-amphotericin), L-glutamine, heparin (sodium salt), and trypsin (0.05%) (Gibco/Life Technologies, Grand Island, NY); fetal bovine serum (HyClone, Orem, Utah); collagenase type I (Worthington Biochemical Corp., Freehold, NJ); endothelial cell growth supplement (ECGS), human recombinant TNFα, FITC-goat anti-mouse IgG, p-nitrophenyl phosphate (PNPP) (Sigma, St. Louis, MO); DiI-labeled acetylated-low density lipoprotein (DiI-Ac-LDL; Biomedical Technologies, Inc., Stoughton, MA); lymphocyte separation medium (Organon Teknika, Durham, NC); anti-ICAM-1 monoclonal antibody (clone LB-2; Becton Dickinson, San Jose, CA); anti-transglutaminase II monoclonal antibody (clone CUB7402; Labvision/Thermo Scientific, Fremont, CA); mouse IgG (Jackson Immunoresearch, West Grove, PA); Background Buster and Immunodiluent (Innovex Biosciences, Richmond, CA); goat anti-mouse IgG-alkaline phosphatase (GAM-AP; BioRad, Hercules, CA).

### Cells

Human umbilical vein endothelial cells (HUVEC) were isolated from umbilical cords using standard methods^44^ or purchased from Lonza (Atlanta, GA). For flow chamber studies, flow cytometry, or binding experiments, HUVEC (passage 2–4) were cultured on 1% gelatin-coated sterile glass slides, 6-well or 96-well culture plates, respectively, until confluent. Human neutrophils were isolated from the citrate-anti-coagulated peripheral blood of healthy volunteers following the sedimentation and hypotonic lysis of erythrocytes^10^, and were re-suspended at 0.5 × 10^6^ cells/ml in M-199 (37 °C) 20 min prior to flow chamber infusion.

### Human subjects

All experiments performed in this study were approved and in accordance with guidelines and regulations as defined by Duke’s Institutional Review Board. For neutrophil isolation, blood was collected from healthy human volunteers via venipuncture after informed consent. Informed consent was obtained from all subjects. After obtaining IRB approval, the umbilical cords that are normally discarded after healthy vaginal deliveries of newborns provided the source for the HUVEC.

#### Expression/purification of TG2 and active site mutant

Purification of wild-type TG2 and active site mutant (TG2/C277A) proteins, the conditions for growing *Escherichia coli* harboring either the TG2 or TG2/C277A vectors, and the purification of glutathione-*S*-transferase (GST) fusion proteins were as previously described^45^. Except as mentioned in the text, GST was cleaved from the purified GST-TG2/C277A fusion protein (called C277A) by incubating with factor Xa (1% w/w, Hematologic Technologies, Inc.) overnight at 4 °C and reapplied to the glutathione resin to remove the cleaved GST protein. Protein concentrations were determined using the Bradford method.

#### Iodoacetamide treatment of TG2 (IODO-TG2)

Recombinant GST-TG2/C277A (1 µM) was incubated with 10 µM of freshly prepared iodoacetamide in buffer containing 50 mM Tris-Cl, pH 7.5, 100 mM NaCl, and 1 mM Ca^2+^ at room temperature for 30 min. After incubation, excess iodoacetamide was removed by dialysis in 4 × 1 liter of buffer containing 50 mM Tris-Cl, pH 7.5, 100 mM NaCl, 1 mM EDTA, and 10% glycerol for 24 hours.

#### Cell treatments

Confluent monolayers of HUVEC were treated with TNFα (10 ng/ml in serum-containing media) for 5 hr prior to experimentation, and during the final hr treated with different concentrations of TG2/C277A in M-199 (3.7 mM Ca^2+^, no serum).

#### Flow chamber

Glass microscope slides with confluent HUVEC monolayers were incorporated into a parallel plate flow chamber. The two halves of the chamber were separated by a 0.005 silicone gasket (Specialty Manufacturing, Inc., Saginaw, MI), which defined the flow path across the endothelial monolayer. Flow medium (M-199 with Hank’s salts, pH 7.4, 37 °C) was pumped through the chamber (#600-000; Harvard Apparatus, Dover, MA) to generate a shear stress (τα) = 0.4 dyn/cm^2^ across the monolayer, which is within the physiologic range estimated in post-capillary venules^46^ and tumor microvessels^47^. The flow chamber was mounted on the stage of an inverted microscope (Zeiss Televal 31, Carl Zeiss, Inc., Thornwood, NY) and the monolayer was visualized via a 10× phase contrast objective (32× overall magnification) and a closed circuit videomicroscopy system, consisting of a Newvicon C2400 tube camera (Hamamatsu Photonics, Hamamatsu, Japan), videotimer (VTG-33; FOR.A, Tokyo, Japan), videotape recorder (SVO-9500MD; Sony, Tokyo, Japan) and monitor (CVM-131, Sony). Following 20 min of preconditioning HUVECs to flow, neutrophils were infused for 5 min, at which time videotaping of an 800 µm × 800 µm area of the monolayer was begun. At 30 sec intervals, the flow rate was increased in stepwise fashion, such that τα = 0.4, 1, 2, and 4 dyn/cm^2^. The range of flow rates used corresponds to Reynolds numbers = 1.5–15.4, indicating the presence of fully developed laminar fluid flow within the flow path. The density of adherent neutrophils was defined as the number of neutrophils remaining stationary (*i.e*, movement <1 cell diameter within 30 sec) per mm^2^ for a given τα. Adherent neutrophils were initially identified at τα = 0.4; subsequently only these adherent neutrophils were followed at higher shear rates. The flux of rolling neutrophils was defined as the number of neutrophils per 30 sec displaying transient interactions with the monolayer past an arbitrarily defined line (200 µm) orthogonal to axis of flow, for postcapillary venular shear stresses (τ_α_ = 0.4 or 1 dyn/cm^2^ only).

#### Shear activation of endothelial cells

HUVEC cells (passage 2–3, Hyclone Laboratories) were grown to confluence on microcarrier beads. TG2/C277A (1 μM) was then added in a final volume of 200 μl and incubated at room temperature for 10 min in a glass cylinder (1 cm in diameter, containing ~3 × 10^7^ cells total) designed to generate rotational shear stress as previously described^22,48^. Recombinant TG2/C277A was recovered from the mixture by low speed centrifugation. Supernatants were store at −80 °C for SNO content determination (Nitrolite and TEA model 543 Analyzer, Thermedics, Woburn, MA) as described^22^.

#### ICAM-1 expression by flow cytometry

HUVECs that had been treated with TNFα and/or TG2/C277A were trypsinized, washed, re-suspended in M-199, and prepared for immunofluorescent staining as previously described^49^. HUVEC were washed (2% BSA, 0.1% NaN_3_ in PBS) without permeabilization, and incubated with either anti-ICAM-1 or mouse IgG (4 °C, 30 min). Following several washes, cells were incubated with FITC-goat anti-mouse IgG (4 °C, 30 min), washed, and fixed (1% paraformaldehyde) for later flow cytometric analysis (Comprehensive Cancer Center Flow Cytometry Lab, Duke University Medical Center).

#### TG2/C277A binding assay

HUVEC were seeded into 96-well plates at 25,000 cells/well, cultured overnight, and then treated with 10 ng/ml TNFα for 5 hrs (37 °C), with 50 nM TG2/C277A added to specified wells. Wells were then washed x3 with HBSS, fixed with 80% methanol (10 min at room temp [RT]), rinsed and incubated with a blocking solution (Background Buster; 10 min, RT). Wells were then incubated with either PBS, mouse IgG, or a TG2 monoclonal antibody (clone CUB7402, 200 µg/ml stock, 1:50 dilution, 30 min, RT) diluted in Immunodiluent. Monolayers were rinsed ×3, incubated with goat anti-mouse-alkaline phosphatase (30 min, RT), and rinsed again. The signal was developed with PNPP and OD readings were determined with a kinetic microtiter plate reader (30 min, RT, 30 sec intervals, 405 nm) (ThermoMax Microplate Reader, Molecular Devices) controlled by SoftMax software.

#### S-Nitrosylation of TG2/C277A *in vitro*

S-nitroso-TG2/C277A (SNO-TG2/C277A) was prepared by incubating TG2/C277A with excess S-nitrosocysteine (in 0.1 mM Tris-acetate, pH 7.5 and 0.1 mM EDTA) at room temperature for 10 min^22^. After S-nitrosylation, free *S*-nitrosocysteine was removed by dialyzing against 4 × 1 liter of buffer containing 50 mM Tris-acetate, pH 7.5, 100 mM NaCl, 10% glycerol at 4 °C^22,50^. SNO content was determined with a photolysis/chemiluminescence detection system (Nitrolite and TEA model 543 Analyzer, Thermedics, Woburn, MA) as described^22^.

#### Immunofluorescent staining

Monolayers of HUVEC were treated with TNFα (10 ng/ml in serum-containing media) for 5 hr prior to staining. Cells were immediately fixed with 4% paraformaldehyde to make membrane non-permeabilize followed by blocking with BSA. TG2 was first incubated with monoclonal antibody against TG2 (TG100) (Labvision, Thermofisher), followed by Donkey anti-mouse IgG conjugated with FITC (Jackson immuno-research) and nuclei was stained with Hoesche 33342.

#### Cell surface protein biotinylation

To label surface expression of endogenous TG2 and exogenous GST-C277A/TG2, HUVEC cells were treated with TNFα for 5 hours and 50 nM of GST-C277A/TG2 cells was added at the final hour. Cell surface proteins were labeled using FluoReporter™ Cell-Surface Biotinylation Kit (ThermoFisher Scientific) on ice according to the instruction manual. Briefly, Biotin-XX sulfosuccinimidyl ester (a cell-impermeant, amine reactive compound) was the active compound that labeled cell surface proteins. After three washes with PBS, cells were lysed with 10 mM HEPES, pH 8, 2 mM EDTA, and 0.5% NP40, protease inhibitors. Total cytoplasmic and membrane fractions were isolated using low speed (500 × G) centrifugation. Biotinylated proteins were isolated on Streptavidin-Dynabead (ThermoFisher Scientific) and analyzed.

#### Resin Assisted Capture for TG2 S-nitrothiols (SNO-RAC)

The analysis of SNO-modified TG2 was carried out with the SNO-RAC method essentially as described^51^. Briefly, C57/B6 mice kidneys were homogenized in lysis buffer (1 g/5 ml lysis buffer) containing 100 mM Hepes/1 mM EDTA/1 mM DTPA/100 mM neocuproine (HEN), 0.1% (vol/vol) Nonidet P-40, a thiol-blocking agent, 0.2% S-methylmethanethiosulfonate (MMTS), and protease inhibitors (Roche). After centrifugation 2X at 20,000 × G for 20 min at 4 °C, SDS was added to the supernatants to 2.5% and incubated at 50 °C for 20 min. Proteins were precipitated with −20 °C acetone, and re-dissolved in 1 mL of HEN/1% SDS. Precipitation of proteins were repeated with −20 °C acetone, and the final pellets were resuspended in HEN/1% SDS and protein concentrations determined using Bicinchoninic Acid (BCA) method. Total lysates (2 mg) were incubated with freshly prepared 50 mM ascorbate/thiopropyl-Sepharose and rotated end-over-end in the dark for 4 h. The bound SNO proteins were sequentially washed with HEN/1% SDS and 0.1X HEN/0.1% SDS, SNO proteins were eluted with 0.1 × HEN/1% SDS/10% β-meracaptoethanol and analyzed by SDS/PAGE and Immunoblotting blotting.

#### Statistical analysis

Comparisons of mean adherent neutrophil density, roller flux, and TG2/C277A-HUVEC binding were tested for differences within each shear stress using Student’s (unpaired) t-tests (*p* < 0.05). GraphPad Prism software was used to analyze the data using a single-factor ANOVA. In addition, posthoc testing using Sidak’s multiple comparisons test to identify the source of statistically significant difference.

## Electronic supplementary material

Supplemental Table and figures
