# Supplementary material for: Endothelial cell-surface tissue transglutaminase inhibits neutrophil adhesion by binding and releasing nitric oxide
Source: Sci Rep. 2017 Nov 23;7:16163. doi: 10.1038/s41598-017-16342-0 (PMC5701052; doi:10.1038/s41598-017-16342-0)
Supplement: Supplementary file 1 — Supplemental Table and figures [file 41598_2017_16342_MOESM1_ESM.pdf]

Supplemental Table and Figures

Endothelial cell-surface tissue transglutaminase inhibits neutrophil adhesion by binding and releasing nitric oxide

Thung-S. Lai[1, 7], Robert A. Lindberg[2], Hua-Lin Zhou, Zishan A. Haroon[3] Mark W. Dewhirst[4], Alfred Hausladen[5], YL, Juang[1], Jonathan S. Stamler[5,6] and Charles S. Greenberg[7] ,

Suppl. Table I. TG2/C277A binds to the surface of endothelial Cells

	W/O	50 nM TG2/C277A
Control	0.053 ± 0.028	0.105 ± 0.017*
TNFα	0.042 ± 0.015	0.089 ± 0.020

Confluent monolayers of HUVEC + TNFα were treated with 50 nM of TG2/C277A as indicated. Non-permeabilized cells were fixed, sequentially incubated with a monoclonal antibody to TG2 followed by an alkaline phosphatase-conjugated secondary antibody. The color was developed by adding substrate PNPP (p-Nitrophenyl Phosphate, Sigma), and read at 405 nm as described under Materials and Methods. Data are presented as mean relative absorbance (anti-TG2 minus IgG ± SEM (n=4 each). * $P < 0.05$ (TG2/C277A *vs* control).

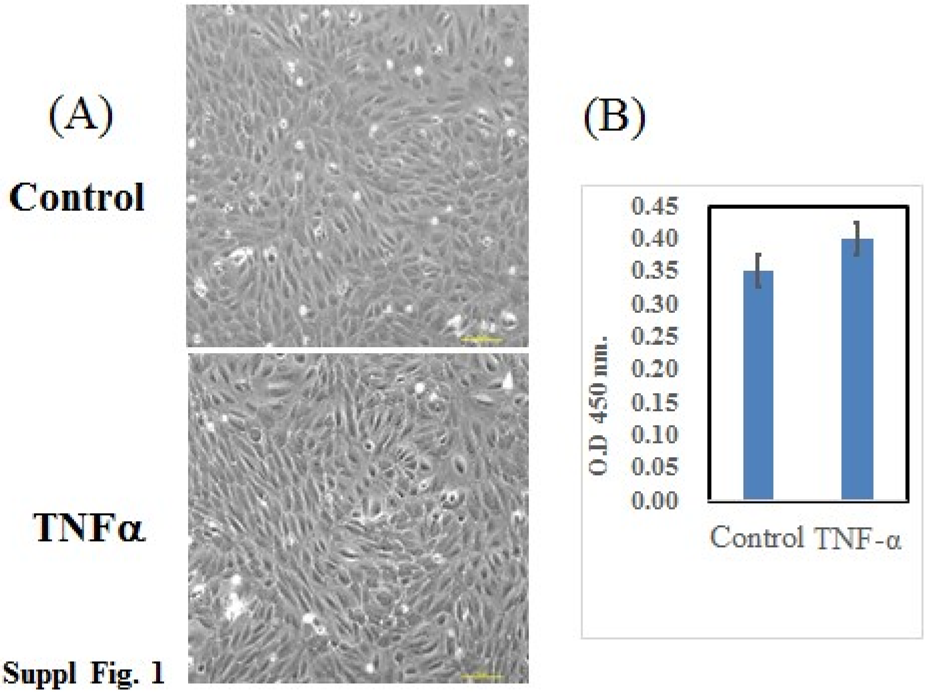

Supplemental Figure 1. Morphology and cell viability after TNFa treatment. HUVEC cells (passage 2-3) were grown to confluence on 6-well dishes and treated with and without 10 ng/ml TNFα for 5 hours in serum containing EGM-2 media. **A**. The morphology of HUVEC cells after 5 hours under light microscopy (200 x). **B.** Cell viability of HUVEC cells after TNFα treatment using Cell Counting Kit (CCK-8, Sigma-Aldrich), according to the instruction manual.

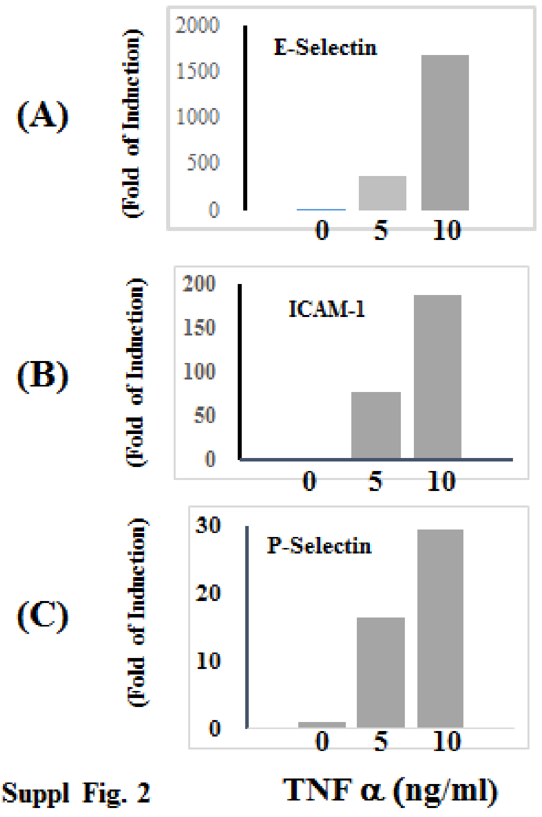

Suppl Fig. 2

Supplemental Figure 2. Quantification of mRNAs encoding E-Selectin, ICAM-1 and P-Selectin. HUVEC cells (passage 2-3) were grown to confluence on 6-well dishes and treated with and without 10 ng/ml TNFα for 5 hours in serum containing EGM-2 media. After TNFα (0, 5 , 10 ng/ml) treatment, total RNAs were isolated and cDNAs were synthesized. Real-time Syber green PCR assays were performed using specific primers as described below and the signals were detected using Applied Biosystem ABI 7900 system. The QPCR primer sequences for human E-Selectin, ICAM-1 and P-selectin are described (1-3). E-Selectin: Forward: 5' GAA GGA TGG ACG CTC AAT GG 3' Reverse: 5' TGG ACT CAG TGG GAG CTT CAC-3'; ICAM-1: Forward: 5'- GCA GAC AGT GAC CAT CTA CAG CTT-3' Reverse:5' CTT CTG AGA CCT CTG GCT TCG T-3'; P-selectin: Forward: 5'- CGC CTG CCT CCA GAC CAT CTT C-3' Reverse: 5'- CTA TTC ACA TTC CAG AAA CTC ACC ACA GC-3'. Amplification was performed using a 94 ^{o}C for 5 min followed by 40 two-step cycle (15 sec 94°C, 1 min 60°C). Melting curves were performed to assure the correct bands were amplified.

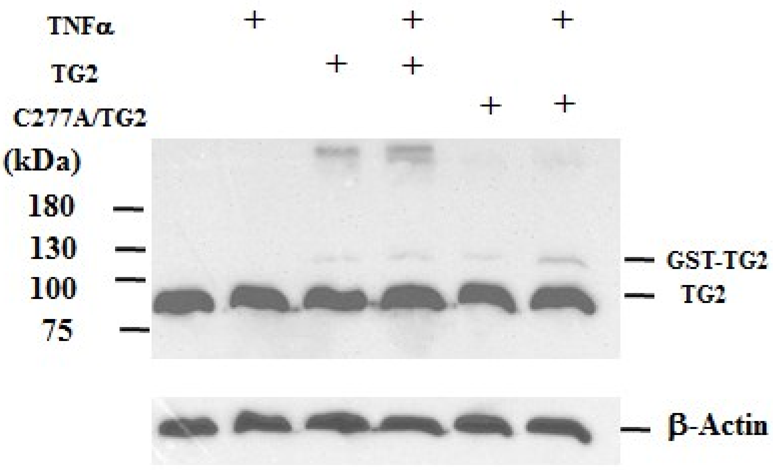

Suppl. Figure 3

Supplemental Figure 3. The binding of TG2 (or TG2/C277A) on the surface of HUVEC cells. HUVEC cells (passage 2-3) were grown to confluence on 6-well dishes and treated with 10 ng/ml TNFα for 5 hours in serum containing EGM-2 media. At the final hour of TNFα treatment, recombinant GST-TG2 (or GST-C277A/TG2, 100 nM each) in EBM2 media (serum free) was added followed by swirling every 10 min. After three washes with serum free EBM-2, cells were lysed with 10 mM HEPES, pH 8, 2 mM EDTA, and 0.5% NP40, protease inhibitors. Total cytoplasmic and membrane fractions were isolated using low speed (500 xG) centrifugation. Ten microgram cell lysates were loaded on a 9 % SDS-PAGE gel and proteins transferred to PVDF membrane. The TG2 related antigens were developed with mouse monoclonal Ab (Cub 7402) against TG2 and color was developed with ECL reagent.

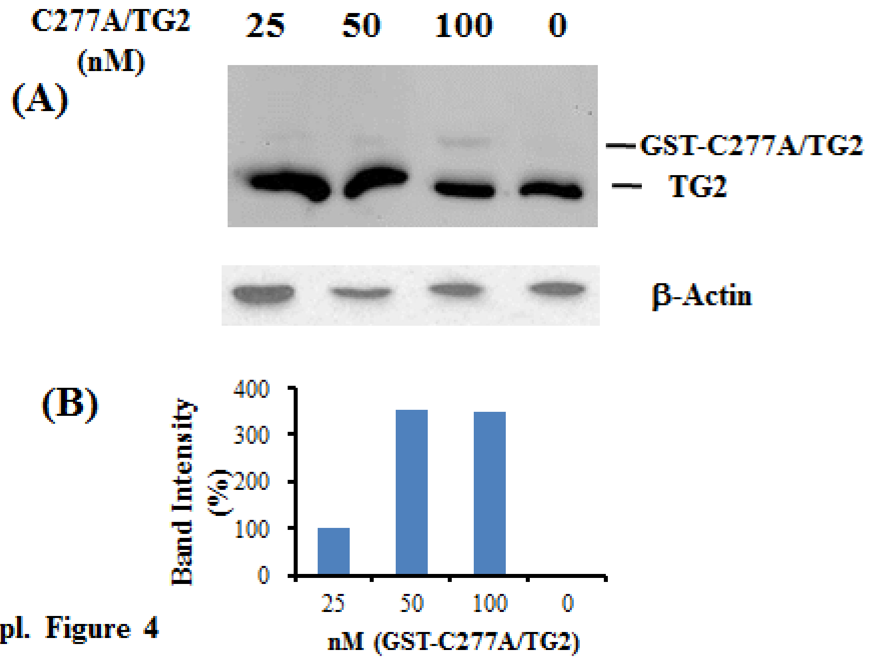

Suppl. Figure 4

Supplemental Figure 4. The dosage-dependent binding of TG2/C277A on the surface of HUVEC cells. HUVEC cells (passage 2-3) were grown to confluence on 6-well dishes and treated with 10 ng/ml TNFα for 5 hours in serum containing EGM-2 media. At the final hour of TNFα treatment, recombinant GST-C277A/TG2, 25, 50 or 100 nM) in EBM2 media (serum free) was added followed by swirling every 10 min. After three washes with serum free EBM-2, cells were lysed with 10 mM HEPES, pH 8, 2 mM EDTA, and 0.5% NP40, protease inhibitors. Total cytoplasmic fractions (including membrane) were isolated using low speed (500 xG) centrifugation. Ten microgram cell lysates were loaded on a 9% SDS-PAGE gel and proteins transferred to PVDF membrane. (A). The TG2 related antigens were detected with mouse monoclonal Ab (Cub 7402) vs TG2 and color was developed with ECL reagent. (B) Densitometry scanning of the GS-C277A/TG2 bands are plotted as percentage increase vs concentrations of GST-C277A/TG2.

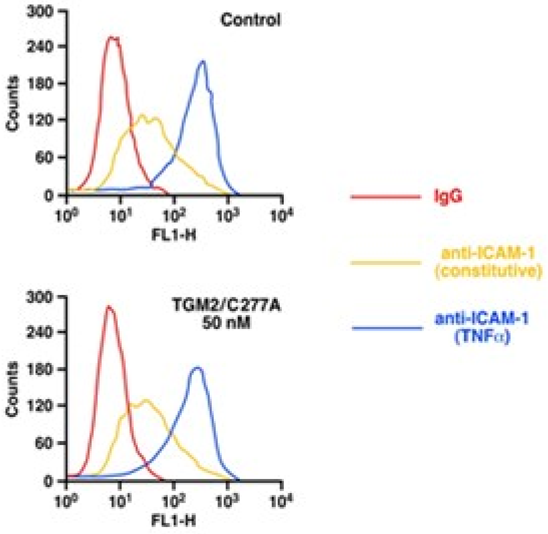

Suppl. Figure 5

Supplemental Figure 5. Expression of ICAM-1 on HUVEC cells treated with TNFα. HUVEC cells treated with 10 ng/ml TNFα and/or TG2/C277A were trypsinized, washed, re-suspended in M-199, and stained as described under Materials and Methods. HUVEC cells were washed (2% BSA, 0.1% NaN_3 in PBS) without permeabilization, and incubated with either anti-ICAM-1 or mouse IgG (4° C, 30 min). Following several washes, cells were incubated with FITC-goat anti-mouse IgG (4° C, 30 min), washed, and fixed (1% paraformaldehyde) for flow cytometric analysis. Red line: IgG control; Yellow line: constitutive ICAM-1; Blue line: TNFα treated ICAM-1.

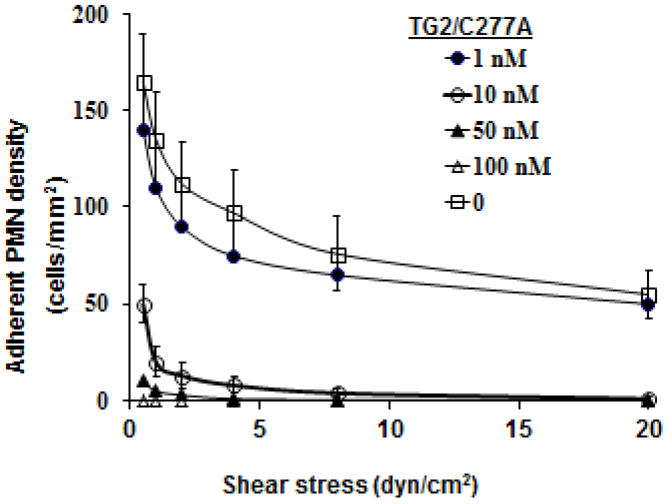

Supplemental Fig. 6

Supplemental Figure 6. TG2/C277A inhibits neutrophil adhesion to TNFα-activated endothelium. HUVEC monolayers were treated with TG2/C277A (different concentrations, 0-100 nM) during the last hour of a 5 hr incubation with TNFα (10 ng/ml). After infusion of neutrophils (PMN), cultures were exposed to shear stress at the values indicated, and adherent PMNs were then counted. Data are presented as means ± SEM. TNFα, n = 5; TNFα/ 10 nM TG2/C277A, n = 4. $p < 0.05$, Statistical difference was analyzed as described under Materials and Methods; Other concentrations of TG2/C277A (1 nM, 50 nM, 100 nM) were done once. TNFα-induced PMN adherence (WO) was significantly reduced upon incubation with 10ng/ml TG2/C277A at each shear stress tested ($p < 0.05$, Student's unpaired 2-sample, 2-tail t-test).

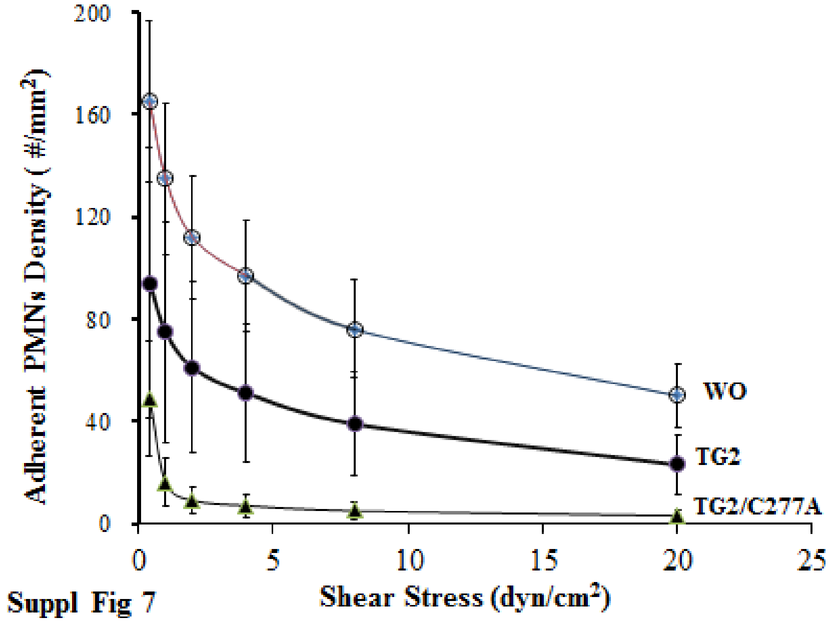

Supplemental Figure 7. Effects of TG2 and TG2/C277A in inhibiting neutrophil adhesion to TNFα-activated endothelium. HUVEC monolayers were treated with TG2 (or TG2/C277A , 10 nM) during the last hr of a 5 hr incubation with TNFα (10 ng/ml). After infusion of neutrophils (PMN), cultures were exposed to shear stress at the values indicated, and adherent PMNs were then counted. Data are presented as means ± SEM. WO, n=5; TG2/C277A, n=4; TG2, n= 4. TNFα-induced PMN adherence (WO) was significantly reduced upon incubation with 10 ng/ml TG2/C277A at each shear stress tested ($p < 0.05$, Student's unpaired 2-sample, 2-tail t-test).

References (Supplemental)

1. Liu, Z., Miner, J. J., Yago, T., Yao, L., Lupu, F., Xia, L., and McEver, R. P. (2010) Differential regulation of human and murine P-selectin expression and function in vivo. *The Journal of experimental medicine* **207**, 2975-2987
2. Laviola, L., Orlando, M. R., Incalza, M. A., Caccioppoli, C., Melchiorre, M., Leonardini, A., Cignarelli, A., Tortosa, F., Labarbuta, R., Martemucci, S., Pacelli, C., Cocco, T., Perrini, S., Natalicchio, A., and Giorgino, F. (2013) TNFalpha signals via p66(Shc) to induce E-Selectin, promote leukocyte transmigration and enhance permeability in human endothelial cells. *PLoS One* **8**, e81930
3. Tu, J., Hu, Z., and Chen, Z. (2013) Endothelial gene expression and molecular changes in response to radiosurgery in in vitro and in vivo models of cerebral arteriovenous malformations. *BioMed research international* **2013**, 408253
